# T4 apoptosis in the acute phase of SARS-CoV-2 infection predicts long COVID

**DOI:** 10.3389/fimmu.2023.1335352

**Published:** 2024-01-03

**Authors:** Renaud Cezar, Lucy Kundura, Sonia André, Claire Lozano, Thierry Vincent, Laurent Muller, Jean-Yves Lefrant, Claire Roger, Pierre-Géraud Claret, Sandra Duvnjak, Paul Loubet, Albert Sotto, Tu-Ahn Tran, Jérôme Estaquier, Pierre Corbeau

**Affiliations:** ^1^ Immunology Department, Nîmes University Hospital, Nîmes, France; ^2^ Institute of Human Genetics, UMR9002, Centre National de la Recherche Scientifique (CNRS) and Montpellier University, Montpellier, France; ^3^ Institut National de la Santé et de la Recherche Médicale (INSERM) U1124, Université de Paris, Paris, France; ^4^ Immunology Department, Montpellier University Hospital, Montpellier, France; ^5^ Surgical Intensive Care Department, Nîmes University Hospital, Nîmes, France; ^6^ Medical and Surgical Emergency Department, Nîmes University Hospital, Nîmes, France; ^7^ Gerontology Department, Nîmes University Hospital, Nîmes, France; ^8^ Infectious Diseases Department, Nîmes University Hospital, Nîmes, France; ^9^ Pediatrics Department, Nîmes University Hospital, Nîmes, France; ^10^ Laval University Research Center, Quebec City, QC, Canada

**Keywords:** programmed cell death, post-acute COVID-19 syndrome, immune activation, helper T lymphocyte, SARS-CoV-2 infection sequelae

## Abstract

**Background:**

As about 10% of patients with COVID-19 present sequelae, it is important to better understand the physiopathology of so-called long COVID.

**Method:**

To this aim, we recruited 29 patients hospitalized for SARS-CoV-2 infection and, by Luminex^®^, quantified 19 soluble factors in their plasma and in the supernatant of their peripheral blood mononuclear cells, including inflammatory and anti-inflammatory cytokines and chemokines, Th1/Th2/Th17 cytokines, and endothelium activation markers. We also measured their T4, T8 and NK differentiation, activation, exhaustion and senescence, T cell apoptosis, and monocyte subpopulations by flow cytometry. We compared these markers between participants who developed long COVID or not one year later.

**Results:**

None of these markers was predictive for sequelae, except programmed T4 cell death. T4 lymphocytes from participants who later presented long COVID were more apoptotic in culture than those of sequelae-free participants at Month 12 (36.9 ± 14.7 vs. 24.2 ± 9.0%, p = 0.016).

**Conclusions:**

Our observation raises the hypothesis that T4 cell death during the acute phase of SARS-CoV-2 infection might pave the way for long COVID. Mechanistically, T4 lymphopenia might favor phenomena that could cause sequelae, including SARS-CoV-2 persistence, reactivation of other viruses, autoimmunity and immune dysregulation. In this scenario, inhibiting T cell apoptosis, for instance, by caspase inhibitors, could prevent long COVID.

## Introduction

In addition to potentially resulting in COVID-19 at its acute phase, SARS-CoV-2 infection may thereafter provoke sequelae, so-called long COVID, in 1 in 10 individuals (1, 2). Long COVID may be defined by the persistence of symptoms, 3 months after the acute infection, for at least 2 months (3). These symptoms encompass asthenia, shortness of breath, neurocognitive disorders, insomnia, anxiety, cardiovascular dysfunction, muscular weakness, and depression (3). As more than three-quarters of a billion cases of COVID-19 have been reported to the World Health Organization (4), post-acute COVID-19 sequelae are a major concern.

One way to address this concern is to better understand the pathophysiology of long COVID by identifying the markers able to predict it. The severity of acute SARS-CoV-2 infection is clearly one of them. Indeed, over 70% of patients hospitalized for COVID-19 presented sequelae (5), particularly those admitted for long stays (6) or to the intensive care unit (ICU) (7). Accordingly, lactate dehydrogenase (LDH), a marker of tissue lesions, has been associated with the development of long COVID (8). C-reactive protein (CRP), Tumor Necrosis Factor-α (TNF- α) (9), interleukin-6 (IL-6), IL-8, CXCL10, peripheral inflammation biomarkers linked to COVID-19 severity, also predict sequelae (8, 10–13), as well as Type I and Type III interferons (IFN) (14). Concerning lymphocytes, the persistence of a low level of naïve T cells (14) and a high level of exhausted T cells (15) was correlated with the presence of sequelae. An increase in inflammatory monocytes was reported in patients who later developed long COVID (16), and CD57-expressing mature/senescent NK cell frequency was identified as predictive for pulmonary sequelae (17).

To further identify the biomarkers predictive of long COVID, we determined the immune profiles of 29 patients recently hospitalized for COVID-19 and monitored them for one year. We then compared the initial immune profile between patients who later developed sequelae or not. Our immune profiling included 19 soluble markers, quantified in plasma and in the supernatant of non-stimulated peripheral blood mononuclear cells (PBMCs), and a panel of cell surface markers analyzing T and NK cell differentiation, activation, exhaustion and senescence, monocyte subpopulations as well as T cell apoptosis. This latter phenomenon is of interest, since we have previously shown that programmed T cell death is linked to disease severity (18). The main form of programmed cell death in the COVID-19 patients we analyzed is apoptosis rather than pyroptosis, necroptosis or PANoptosis, since transcripts of the proapoptotic Bcl-2 family members, Bax and Bak, were up-regulated in T cells, CD95 was over-expressed at the T cell surface, plasma level of soluble Fas was increased, and the pan-caspase inhibitor Q-VD was more efficient in preventing T cell death than the inflammasome/pyroptosis inhibitors VX765, IDN6556, and MCC950 and the necroptosis inhibitors GSK-872 and Dafrafenib (18).

## Materials and methods

### Study design

Volunteers were recruited between April and October 2020 on the very first day of their hospitalization in the intensive care unit (ICU) or non-ICU at Nîmes University Hospital, France, for SARS-CoV-2 infection confirmed by RT-PCR performed on a nasopharyngeal swab. All participants provided written informed consent and the study was approved by the Île-de-France 1 Ethics Committee. The trial was registered on ClinicalTrials.gov under the reference NCT04351711.

### Flow cytometry

Conjugated monoclonal antibodies purchased from Beckman Coulter were used in the following combinations; CD57-fluorescein isothiocyanate (FITC)/CD279-phycoerythrin (PE)/CD45RA- energy-coupled dye (ECD)/CD8-allophycocyanine (APC)/CD4- APC/Alexa700 (APC700)/CD3-APC/Alexa750 (APC750), CD8-APC/CD4-APC700/CD3-APC750/CD38-PE/HLADR-PE-Cyanine7 (PC7), CD3-APC750/CD16-APC/HLADR-PC7/CD56- PE-Cyanine5.5 (PC5.5)/CD57-FITC, CD14-PE/CD16-APC.

Whole blood collected on EDTA was labeled and stored in the dark for 10 minutes at room temperature and fixed (IMMUNOPREP reagent system kit and TQ Prep automate, Beckman Coulter) according to the manufacturer’s recommendations. Samples were run on a Navios flow cytometer (Beckman Coulter) and results were analyzed using Kaluza software (Beckman Coulter). A minimum of 20,000 lymphocytes were analyzed per subpopulation. The flow cytometer’s inter-run stability was verified using the same batch of FlowSet Pro Beads (Beckman Coulter).

### Soluble immune activation markers

We used TruCulture tubes (Myriad RBM) which allow blood collection and culture in a single tube. To analyze the soluble factors spontaneously released by PBMCs in culture, we chose TruCulture tubes devoid of immune stimulant. After drawing 1mL of blood, the tube was inserted into a dry block incubator maintained at 37°C for 48 h. The tube was then opened, and culture supernatant was separated from the sedimented cells and maintained at −20°C.

IL-1α, IL-1β, IL-4, IL-6, IL-8, IL-10, IL-12p70, IL-13, IL-17A, GM-CSF, IFN-α, IFN-γ, MIP-1α, MIP-1β, TNF-α, IP-10/CXCL10, soluble ICAM-1, soluble E-selectin, and soluble P-selectin were quantified in plasmas and PBMC supernatants by Luminex/xMAP immunoassays (ProcartaPlex, ThermoFisher scientific) according to the manufacturer’s instructions.

### Statistical analysis

The d’Agostino and Pearson test was used to assess normality. Group comparisons were performed with a two-sided unpaired Student t-test or Mann-Whitney test as appropriate. Adjusted p-values were calculated using the Benjamini-Krieger-Yekutieli method when necessary. A p-value < 0.05 was considered as statistically significant.

## Results

### Patient characteristics

We recruited 29 PCR-positive SARS-CoV-2-infected patients on the very first day of admission to the ICU or the Infectious Diseases Department (non-ICU) at Nîmes University Hospital, France. ICU patients presented an oxygen saturation of less than 90% and/or PaO2 below 60 mmHg in room air, or an oxygen saturation of less than 90% while receiving 5L/min of oxygen. Non-ICU patients had an oxygen saturation of less than 96% in room air and/or deterioration in their general condition. The bioclinical characteristics of these patients during the acute phase of the infection are shown in **Table 1**. These 29 volunteers were monitored over a year. At Month 12, 19 (66%) of them presented long COVID. Sequelae included asthenia (58%), neurologic disorders (42%), breathing difficulties (37%), insomnia (37%), anxiety (21%), cardiovascular disorders (16%), muscular weakness (10%) and depression (10%). In line with the literature (5–7), the severity of COVID-19 was a risk factor for developing post-acute syndrome. Thus, the incidence of long COVID was 11-fold higher in ICU than in non-ICU patients (**Table 1**). Likewise, individuals with long COVID tended to have been hospitalized for a longer period than those without post-acute syndrome (mean ± SD, 9.6 ± 6.9 vs. 5.9 ± 6.6 days). As previously reported (8), biomarkers of severity, like high lactate dehydrogenase concentration in plasma (mean ± SD, 381 ± 147 vs. 195 ± 53 U/L) and low levels of circulating monocytes (mean ± SD, 0.42 ± 0.22 vs. 0.78 ± 0.33 x 10^9^/L), were also associated with the development of long COVID.

**Table 1:** Characteristics of the study volunteers at the acute phase of the infection.

		Long COVID Patients (n=19)	Non- Long COVID patients (n=10)	Long COVID vs. non-Long COVID (p-value)
Age (years)	Mean ± SD Range	68.5 ± 15.3 39-94	58.4 ± 20.8 29-92	0.243
Gender: Females Males	% %	38 62	30 70	0.697
Intensive care unit Non-intensive care unit	% %	92 44	8 56	**0.008**
Diabetes	%	21	30	0.665
Duration of hospitalization (days)	Mean ± SD	9.6 ± 6.9	5.9 ± 6.6	0.059
C-reactive protein (mg/L, normal range 0.9-1.8))	Mean± SD	78.2 ± 73.8	56.4 ± 53.4	0.478
Lactate dehydrogenase (U/L, normal range 0.9-1.8)	Mean ± SD	381 ± 147	195 ± 53	**0.006**
Absolute lymphocyte count (x10^9^/L, normal range 0.9-1.8)	Mean ± SD	0.97 ± 0.43	1.34 ± 0.72	0.174
Absolute monocyte count (x10^9^/L, normal range 0.9-1.8)	Mean (SD)	0.42 ± 0.22	0.78 ± 0.33	**0.003**

Bold p-values are below or equal to 0.05.

### Immune activation in the acute phase of infection: soluble markers

As it has been reported that patients who develop long COVID present more marks of immune activation during the acute phase of their infection than patients who do not (8, 10–17), we measured the plasma levels of a panel of soluble markers on the first day of hospitalization in the 29 volunteers recruited. We chose to measure inflammatory cytokines IFN-α, IFN-γ, TNF-α, IL-1α, IL-1β, IL-6, IL-8, anti-inflammatory cytokine IL-10, TH1 cytokine IL-12, TH2 cytokines IL-4 and IL-13, TH17 cytokine IL-17A, growth factor GM-CSF, inflammatory chemokines IP-10, MIP-1α and MIP-1β, as well as the endothelium activation markers ICAM-1, E- and P-selectins. Comparing the plasma levels of these markers during the acute SARS-CoV-2 infection in individuals who later presented long COVID or not, we observed no difference (**Table 2**). We quantified the same markers in the supernatant of PBMC cultured for 48 hours without stimulation. **Table 2** shows that the PBMC of patients with sequelae initially spontaneously released higher amounts of the soluble endothelium activation marker P-selectin. Yet, after false discovery rate correction, this difference did not appear significant.

**Table 2:** Soluble activation marker concentrations in plasma and PBMC supernatant in the acute phase of infection in patients who suffered from long COVID one year later or not.

			Long COVID patients (n=19)	Non- long COVID patients (n=10)	Long COVID vs. non- Long COVID p-value	Long COVID vs. non- Long COVID adjusted-p-value
Plasma		Units	Means (SD)	Means (SD)		
	GM-CSF	pg/mL	47.5 (36.0)	21.9 (22.2)	0.069	NS
	Soluble ICAM-1	pg/mL	29,712 (15,081)	31,029 (25,675)	0.892	NS
	IFN-gamma	pg/mL	17.8 (12.3)	12.0 (9.8)	0.258	NS
	IFN-alpha	pg/mL	6.27 (4.8)	7.2 (6.4)	0.715	NS
	IL-1alpha	pg/mL	5.27 (10.6)	1.29 (1.84)	0.308	NS
	IL-1beta	pg/mL	87.7 (75.2)	68.4 (56.0)	0.804	NS
	IL-10	pg/mL	8.03 (5.06)	4.69 (3.47)	0.099	NS
	IL-12	pg/mL	41.8 (61.3)	14.0 (9.8)	0.210	NS
	IL-13	pg/mL	18.1 (8.0)	10.3 (4.5)	0.057	NS
	IL-17A	pg/mL	18.9 (16.6)	29.2 (28.8)	0.340	NS
	IL-4	pg/mL	32.2 (24.8)	15.1 (15.6)	0.078	NS
	IL-8	pg/mL	15.7 (43.3)	3.1 (5.4)	0.507	NS
	IP-10	pg/mL	39.5 (19.5)	48.8 (59.3)	0.497	NS
	IL-6	pg/mL	71.4 (75.6)	139.5 (187.6)	0.305	NS
	MIP-1alpha	pg/mL	16.9 (16.2)	44.5 (75.9)	0.263	NS
	MIP-1beta	pg/mL	25.4 (30.8)	6.6 (12.4)	0.197	NS
	Soluble E-Selectin	pg/mL	8,231 (6,429)	11,585 (5,703)	0.215	NS
	Soluble P-Selectin	pg/mL	1,027,455 (3,324,110)	93,777 (56,707)	0.549	NS
	TNF-alpha	pg/mL	101.9 (277.6)	22.9 (12.2)	0.379	NS
Supernatant of non-stimulated PBMC	GM-CSF	pg/mL	1.04 (3.08)	4.44 (9.36)	0.321	NS
	Soluble ICAM-1	pg/mL	138,707 (129,291)	54,504 (21,873)	0.277	NS
	IFN-gamma	pg/mL	8.77 (9.18)	9.94 (16.35)	0.413	NS
	IFN-alpha	pg/mL	4.18 (5.66)	9.54 (15.82)	0.531	NS
	IL-1alpha	pg/mL	12.30 (16.87)	15.98 (18.14)	0.494	NS
	IL-1beta	pg/mL	24.5 (39.5)	22.8 (34.6)	0.529	NS
	IL-10	pg/mL	1.46 (2.07)	0.67 (1.32)	0.182	NS
	IL-12	pg/mL	47.3 (64.4)	14.3 (29.0)	0.141	NS
	IL-13	pg/mL	8.38 (4.38)	6.73 (4.71)	0.359	NS
	IL-17A	pg/mL	17.3 (28.7)	64.0 (139.9)	0.265	NS
	IL-4	pg/mL	21.8 (14.7)	13.2 (17.1)	0.176	NS
	IL-8	pg/mL	531.6 (1,035.0)	251.2 (323.0)	0.981	NS
	IP-10	pg/mL	73.7 (86.4)	26.4 (27.2)	0.133	NS
	IL-6	pg/mL	147.9 (342.2)	229.7 (481.2)	0.096	NS
	MIP-1alpha	pg/mL	19.3 (19.6)	52.8 (92.1)	0.382	NS
	MIP-1beta	pg/mL	260.8 (347.9)	138.8 (129.7)	0.524	NS
	Soluble E-Selectin	pg/mL	12,968 (9,019)	11,810 (5,666)	0.981	NS
	Soluble P-Selectin	pg/mL	351,157 (577,864)	117,298 (72,502)	**0.031**	0.463
	TNF-alpha	pg/mL	22.4 (24.6)	13.8 (19.7)	0.350	NS

Bold p-values are below or equal to 0.05. NS, not significant.

### Immune activation in the acute phase of infection: cell surface markers

**Figure 1:**
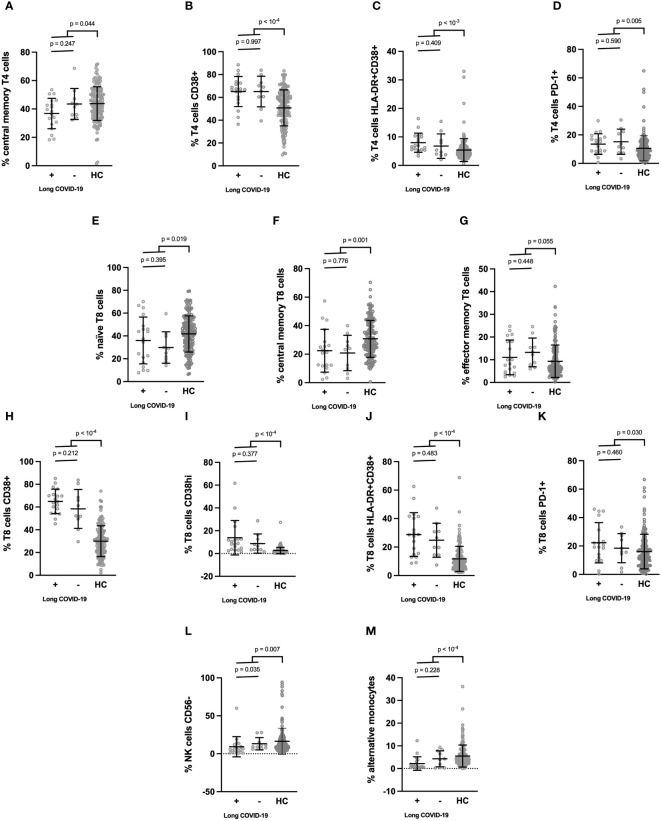
**(A–M)** Cell surface activation markers which differed between patients in the acute phase of SARS-CoV-2 infection and healthy donors. Patients were split into those who developed long COVID one year later and those who did not. Means ± SD are indicated.

Next, still looking for differences between participants who were later afflicted by sequelae or not, we analyzed markers on the surface of immune cells in the acute phase of infection (**Table 3**). To this end, we determined the differentiation (naïve vs. central memory vs. effector memory cells), activation (HLA-DR+, CD38+), exhaustion (PD-1+), senescence (CD57+) of T4 and T8 lymphocytes, the differentiation (CD56 expression), activation (HLA-DR+), maturation/senescence (CD57 expression) of NK cells, and the proportions of monocyte subpopulations (CD14+CD16low classical, CD14+CD16+ intermediate, CD14lowCD16+ alternative). The same markers were studied in 150 age- and sex-matched healthy controls (HCs). Compared to HCs, COVID-19 patients were characterized by a low level of central memory T4 cells (**Figure 1A**) and high percentages of activated (CD38+ and HLA-DR+CD38+, **Figures 1B, C**) and exhausted (PD-1+, **Figure 1D**) T4 cells. Yet, after false discovery rate adjustment, only the increase in T4 activation remained significant (**Table 3**). Concerning COVID-19 T8 lymphocytes, we noticed a differentiation (decrease in naïve and central memory cells contrasting with an increase in effector memory cells, **Figures 1E–G**), activation (CD38+, CD38hi, and HLA-DR+CD38+, **Figures 1H–J**), and exhaustion (PD-1+, **Figure 1K**). Yet, regarding the increase in PD-1+ T8 cells, the adjusted-p-value was above 0.05 (**Table 3**). CD16-negative NK cells appeared diminished in SARS-CoV-2 infected individuals after p-value adjustment (**Figure 1L**; **Table 3**). Finally, monocytes showed a decrease in the alternative CD14lowCD16+ subpopulation (**Figure 1M**). Yet, we found no difference in these various leucocyte subsets between patients who suffered long-term effects or not (**Table 3**).

**Table 3:** Cell surface activation markers in patients in the acute phase of infection who suffered from long COVID one year later or not, and in healthy donors.

Biomarker	Long COVID patients (n=19)	Non- long COVID patients (n=10)	Healthy Controls (n = 151)	Long COVID vs. non-Long COVID p-value (adjusted p-value)	COVID vs. healthy controls p value (adjusted p-value)
% naïve T4 cells	42.6 ± 15.6	42.6 ± 15.4	41.8 ± 15.9	0.999	0.793
% effector memory T4 cells	12.5 ± 8.0	11.5 ± 5.4	11.5 ± 9.1	0.712	0.693
**% central memory T4 cells**	38.3 ± 10.2	43.5 ± 10.9	43.7 ± 11.9	0.247	**0.044** (NS)
% T4 cells HLA-DR+	13.2 ± 5.5	14.0 ± 10.0	15.9 ± 11.1	0.765	0.251
**% T4 cells CD38+**	65.1 ± 13.2	65.1 ± 13.4	50.8 ± 15.8	0.997	**< 10^-4^ (<10^-4^)**
**% T4 cells HLA-DR+CD38+**	8.0 ± 3.3	6.8 ± 4.3	5.4 ± 4.0	0.409	**< 0.001 (0.004)**
% T4 cells CD57+	8.9 ± 10.8	4.4 ± 2.9	5.9 ± 7.8	0.875	0.539
**% T4 cells PD-1+**	13.5 ± 7.2	15.2 ± 8.9	10.6 ± 8.8	0.590	**0.045** (NS)
**% naïve T8 cells**	36.1 ± 20.5	29.8 ± 13.8	41.8 ± 15.9	0.395	**0.019 (0.041)**
**% effector memory T8 cells**	11.0 ± 7.6	13.2 ± 6.3	9.3 ± 7.1	0.448	0.055
**% central memory T8 cells**	22.4 ± 15.0	20.8 ± 12.4	30.7 ± 13.0	0.776	**0.001 (0.004)**
% T8 cells HLA-DR+	36.4 ± 17.0	38.0 ± 15.5	39.6 ± 17.5	0.821	0.480
**% T8 cells CD38+**	64.9 ± 10.8	58.3 ± 17.1	30.0 ± 13.5	0.212	**< 10^-4^ (< 10^-4^)**
**% T8 cells CD38hi**	12.4 ± 14.6	8.7 ± 8.5	2.7 ± 2.8	0.377	**< 10^-4^ (< 10^-4^)**
**% T8 cells HLA-DR+CD38+**	28.8 ± 15.3	24.8 ± 12.0	11.7 ± 8.8	0.483	**< 10^-4^ (< 10^-4^)**
% T8 cells CD57+	30.3 ± 22.0	35.4 ± 17.5	29.2 ± 16.4	0.266	0.696
**% T8 cells PD-1+**	21.0 ± 13.9	18.4 ± 10.2	16.0 ± 12.2	0.460	0.030 (NS)
**% NK cells CD56-**	9.22 ± 13.3	13.2 ± 8.2	16.4 ± 17.1	0.035 (NS)	**0.007 (0.018)**
% NK cells HLA-DR+	24.0 ± 22.2	17.3 ± 19.4	16.4 ± 12.3	0.286	0.472
% NK cells CD57+	44.9 ± 14.7	51.6 ± 11.7	49.2 ± 16.4	0.320	0.351
% classical monocytes	85.5 ± 9.3	80.1 ± 12.7	79.9 ± 14.4	0.204	0.125
% intermediate monocytes	12.3 ± 8.5	15.6 ± 11.4	10.0 ± 6.7	0.385	0.104
**% alternative monocytes**	2.2 ± 3.0	4.3 ± 3.5	5.5 ± 4.8	0.228	**< 10^-4^ (< 10^-4^)**

Data are represented as Means ± SD.

Bold p-values are below or equal to 0.05. NS, not significant.

### T4 apoptosis in the acute phase of the infection predicts sequelae

**Figure 2:**
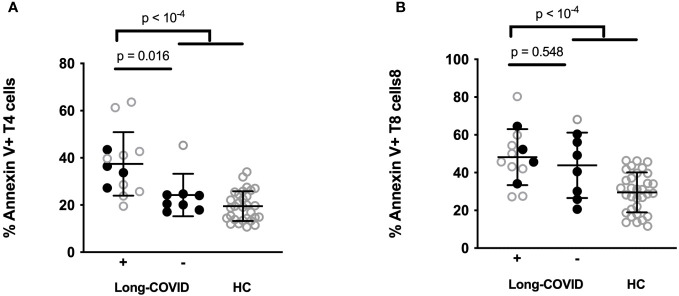
Percentages of CD4+ **(A)** and CD8+ **(B)** T cells expressing Annexin V in ICU (open circles) and non-ICU (closed circles) patients acutely infected by SARS-CoV-2 who later developed sequelae (Long COVID +) or those who did not (Long COVID -) and in healthy controls (HC).

We had previously observed that programmed T cell death was a characteristic of severe COVID-19 (18, 19). The destruction of T cells during acute infection might pave the way for persistence of SARS-CoV-2, reactivation of other viruses such as cytomegalovirus (CMV) and Epstein-Barr virus (EBV), autoimmunity, and immune dysregulation, phenomena which are all thought to potentially provoke long COVID. We therefore explored the hypothesis that T cell apoptosis might favor sequelae. For this purpose, we tested whether the intensity of T4 and T8 apoptosis that we measured in 21 patients at the acute phase of infection predicted sequelae. As we previously reported, compared with the healthy state, the acute phase of infection was characterized by a high level of T4 (31.6 ± 13.9 vs. 19.5 ± 6.3%, p < 10^-4^) and T8 (46.5 ± 15.5 vs. 29.5 ± 10.6%, p < 10^-4^) apoptosis (**Figure 2**). Interestingly, we noted that long COVID was preceded by a high level of programmed T4, but not T8, cell death at the acute phase (**Figure 2**). Indeed, patients who later suffered sequelae presented a higher level of T4 apoptosis than those who did not (36.9 ± 14.7 vs. 24.2 ± 9.0%, p = 0.016). This was particularly obvious for non-ICU patients, with a threshold of 25% T4 apoptosis distinguishing individuals doomed to suffer sequelae from individuals without (**Figure 2A**). Nonetheless, this was not the case for T8 apoptosis (46.2 ± 16.4 vs. 43.9 ± 17.34%, p = 0.611) which was similar between participants who developed post-acute syndrome or not (**Figure 2B**).

## Discussion

In the present study of 29 individuals hospitalized for acute SARS-CoV-2 infection, we measured a large panel of biomarkers in order to unveil those capable of predicting the subsequent onset of long COVID. As a non-significant predictor, we identified the peripheral blood level of soluble P-selectin, a marker of endothelium and platelet activation, already positively associated with severe COVID-19 (20). Nonetheless, we did not identify any inflammation markers previously linked to the onset of sequelae, such as TNF-α, IL-6 or IFNI, as predictors of long COVID (8–14). This may be explained by the limited number of patients in our cohort.

However, we did identify an increase in T cell differentiation, activation, and exhaustion as well as perturbations in and NK monocyte subpopulations in patients with severe COVID-19 as observed by many groups (21–25). But these markers of COVID-19 severity did not differentiate patients who progressed towards long COVID from those who did not. Here again, the low number of patients we analyzed may have masked differences.

By contrast, the intensity of programmed T4 cell death in the acute phase of the infection was able to perform this differentiation. Note that the relatively small number of participants in this study emphasizes the strength of this link between initial T4 cell death and subsequent sequelae.

In a previous work, we showed that T cell apoptosis was a major feature of severe COVID-19, more frequent in ICU than in non-ICU patients, and accounting for lymphopenia (18). One consequence of T4 apoptosis is the poor quality of the anti-spike humoral response from patients with critical disease (26). Given the ability of cytotoxic T cells to kill activated immune cells in the course of viral infections, T8 apoptosis might also favor a cytokine storm (27). Our data now raises the additional hypothesis that T4 apoptosis might be a cause of sequelae in SARS-CoV-2 infection.

Various pathophysiological hypotheses have been proposed to explain long COVID (28, 29). First, sequelae could be the consequence of initial tissue lesions. A second potential cause is viral, either persistence of SARS-CoV-2 or reactivation of other viruses such as EBV. A third category of etiology is immunological dysfunction, in particular autoimmunity and ongoing immune activation. Endothelial activation and coagulopathy have also been proposed. Finally, some authors argue for a role of dysbiosis or metabolic and hormonal dysregulation. Note that many of these putative drivers may be fueled by T4 apoptosis. Programmed CD4+ T-lymphocyte death at the peak of infection might hinder specific immune responses and thereby SARS-CoV-2 eradication, favoring tissue lesions. It may also facilitate the reactivation of latent viruses. In addition, T4 deficiency is known to provoke immune activation and autoimmunity (30). Immune activation is also known to provoke endothelial cell activation and coagulation (31). Finally, given the interactions between, on the one hand, the immune system and microbiota (32), metabolism (33), as well as hormones (34) on the other, T4 loss may provoke dysbiosis, hormonal and metabolic perturbations. Thus, in addition to impairing anti-SARS-CoV-2 immune response and participating in a cytokine storm during the acute phase, programmed T4 cell death could thereafter contribute to sequelae.

We recently reported that a low frequency of perforin-positive T8 cells may be observed at the acute phase of SARS-CoV-2 infection in patients who later presented sequelae. This observation aligns with our present data, arguing for a role of T cell deficiency in long COVID.

Our results are a supplementary argument for trying to prevent T cell apoptosis in SARS-CoV-2 infection. In this perspective, we have also shown that the pan-caspase inhibitor Q-VD inhibited ex vivo programmed death of COVID-19 T cells (18). Moreover, we have also reported that angiotensin II-induced monocytic reactive oxygen species production caused DNA damage and T cell apoptosis in hospitalized patients, a cascade which may be blocked by an angiotensin-II-receptor antagonist (sartan) and the antioxidant N-acetylcysteine (19). Therefore, sartans and N-acetylcysteine might be good candidates for impeding T cell apoptosis in the course of SARS-CoV-2 infection and the development of long COVID.

## Data availability statement

The original contributions presented in the study are included in the article/supplementary material. Further inquiries can be directed to the corresponding authors.

## Ethics statement

The studies involving humans were approved by Île-de-France 1 Ethics Committee. The studies were conducted in accordance with the local legislation and institutional requirements. The participants provided their written informed consent to participate in this study.

## Author contributions

RC: Data curation, Formal analysis, Investigation, Methodology, Writing – review & editing. LK: Data curation, Formal analysis, Investigation, Methodology, Writing – review & editing. SA: Investigation, Resources, Supervision, Validation, Writing – review & editing. CL: Data curation, Formal analysis, Methodology, Supervision, Validation, Writing – review & editing. TV: Resources, Supervision, Validation, Writing – review & editing. LM: Investigation, Resources, Supervision, Validation, Writing – review & editing. J-YL: Investigation, Resources, Supervision, Validation, Writing – review & editing. CR: Investigation, Resources, Supervision, Validation, Writing – review & editing. P-GC: Investigation, Resources, Supervision, Validation, Writing – review & editing. SD: Resources, Supervision, Validation, Writing – review & editing, Investigation. PL: Investigation, Resources, Supervision, Validation, Writing – review & editing. AS: Investigation, Resources, Supervision, Validation, Writing – review & editing. T-AT: Investigation, Resources, Supervision, Validation, Writing – review & editing. JE: Investigation, Resources, Supervision, Validation, Writing – review & editing. PC: Conceptualization, Data curation, Funding acquisition, Methodology, Project administration, Resources, Supervision, Validation, Writing – original draft.
